# The role of interleukin‑7 serum level as biological marker in breast cancer: a cross‑sectional, observational, and analytical study

**DOI:** 10.1186/s12957-022-02646-7

**Published:** 2022-07-06

**Authors:** Faton Sermaxhaj, Natalija Dedić Plavetić, Ugur Gozalan, Ana Kulić, Ljubica Radmilović Varga, Marina Popović, Slavica Sović, Davor Mijatović, Besim Sermaxhaj, Mentor Sopjani

**Affiliations:** 1 Clinic of Oncology, University Clinical Center of Kosovo, Prishtina, Kosovo; 2 Department of Oncology, University Hospital Centre Zagreb, Zagreb, Croatia; 3 School of Medicine, University of Zagreb, Zagreb, Croatia; 4 Department of General Surgery, Royal Hospital, Prishtina, Kosovo; 5 Department of Pulmonology, Varazdin General Hospital, Varaždin, Croatia; 6 Department of Plastic Surgery, University Hospital Centre Zagreb, Zagreb, Croatia; 7 Department of Surgery, University Clinical Center of Kosovo, Prishtina, Kosovo; 8 Faculty of Medicine, University of Prishtina, Prishtina, Kosovo

**Keywords:** Breast cancer, Interleukins, Interleukin-7, Kosovo, Croatia, Biomarker

## Abstract

**Background:**

The important role that the immune system plays in malignant diseases is well known. The action of interleukin-7 (IL-7) as a cytokine has been observed in many cellular processes, both in normal cells of the immune system and in some cancer cells. The aim of this study has been to explore whether there is any elevation of interleukin-7 serum levels in early invasive breast cancer (EIBC) patients in comparison with healthy controls. In addition, the correlation between the IL-7 serum level and the histopathological characteristics of the tumor has been evaluated.

**Methods:**

This cross-sectional, observational, and analytical study included 213 consecutive patients with EIBC (113 from Croatia and 100 from Kosovo) and 62 healthy participants as the control group (30 from Croatia and 32 from Kosovo). Blood samples have been taken from patients confirmed with breast cancer (BC) by biopsy, prior to surgical intervention and other oncological treatments, as well as from healthy participants. A serum IL-7 level has been measured, using the “Sandwich” ELISA Immunoenzyme test. In addition, after the surgical intervention, histopathological specimen examinations and immunohistochemistry have been performed and analyzed. The differences in the distribution of the numerical variables have been analyzed with the Mann–Whitney *U* test and Kruskal–Wallis ANOVA test. Correlations have been tested with Pearson coefficients. A *P*-value < 0.05 has been accepted as statistically significant.

**Results:**

The serum level of IL-7 in EIBC patients was significantly higher than in control cases (*P* 0.001). Patients with invasive lobular carcinoma (ILC) seem to have a lower IL-7 serum level compared to other histological subtypes, and the difference has been significant (*P* = 0.043). There has been no correlation between IL-7 serum level and histopathological characteristics of the tumor, with neither age nor menopausal status of the patients.

**Conclusions:**

Noting the significant increase in the IL-7 serum level in the EIBC patients as compared to the healthy control group, the use of IL-7 as a potential diagnostic indicator for BC, as well as in the follow-up of the patients after treatment, can be assumed. The lack of correlation with tumor size, lymph node metastasis, and all other histopathological characteristics of the tumor questions its use as a prognostic indicator.

## Introduction

BC is a common disease worldwide with an increasing incidence every year (2,261,419 new cases for the year 2020), and it is responsible for thousands of deaths annually (684,996 for the year 2020) [1]. An early and accurate diagnosis is a prerequisite to effective treatment and the survival of the patient. Tumor markers play an important role in clinical practice for screening, disease staging, prognosis, and evaluation of treatment effectiveness in some types of malignancies. Although there are tumor markers such as CEA and CA15-3, which are in use for the follow-up of metastatic BC patients, the investigation of an ideal tumor marker is continuing.

The human body’s immune system plays an important role in malignant diseases, first identifying malignant cells and then fighting them. In the early stages of tumor progression, immune cells such as natural killer (NK) and CD8^+^T cells identify and destroy most of the cancer cells [2]. Much research has already been done on different cytokines in order to evaluate their role in the development of various tumors. Recently, the possibility of using different cytokines for the treatment of tumors has been considered [3, 4].

**Fig. 1:**
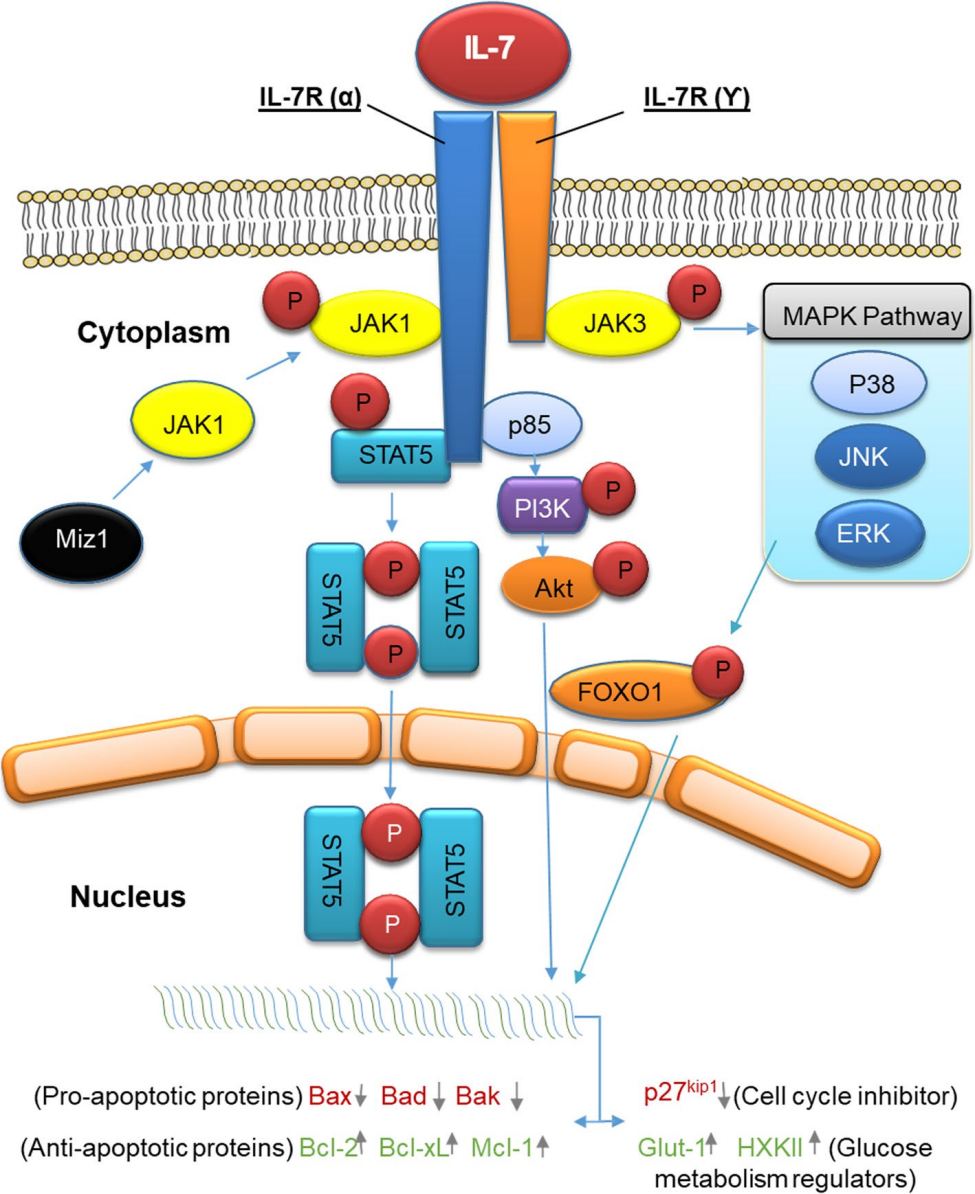
IL-7 induced intracellular signaling pathways. The non-hematopoietic cell-derived cytokine IL-7 has to combine with the IL-7R on the cell membrane to realize its biological activity. IL-7R complex is a transmembrane heterodimer consisting of two subunits, the ligand-binding IL-7 receptor α chain and the common signal-transducing γ chain (γc chain), which is shared by IL-7R, IL-2R, IL-4R, IL-9R, IL-15R, and IL-21R. JAK/STAT, PI3K-Akt, and MAPK signaling pathways are involved and have specific roles in IL-7-mediated functions. Upon IL-7 binding to its receptor, IL-7Rα, the α and γc subunits dimerize. This induces intracellular, non-receptor tyrosine kinases, Janus kinases (JAK) 1 and 3, to activate and mediate the IL-7 signaling transduction. To this end, the transcription factor Miz1 (Myc-interacting zinc finger protein 1) recruits JAK1 to IL-7Rα. This induces phosphorylation of JAK1 and JAK3, which in turn triggers STAT5 phosphorylation with subsequent dimerization to regulate gene expression, i.e., stimulate anti-apoptotic gene expression and inhibit pro-apoptotic gene expression. On the other hand, JAK1 and JAK3 phosphorylation leads to the activation of MAPK and PI3K-Akt signaling pathway as presented in the figure

IL-7 is a cytokine of particular importance to the immune system. It supports the development of lymphocytes in the thymus and the organogenesis of the lymph nodes, and it ensures the maintenance of activated T cells in the secondary lymphoid organs [5, 6]. Non-hematopoietic cells that produce IL-7 include fibroblastic cells in the bone marrow and lymphoid organs; epithelial cells in the thymus, prostate, and intestine [7–9]; and keratinocytes in the skin [10]. The production of IL-7 by dendritic cells of the immune system has also been observed [11]. On the other hand, evidence shows that IL-7 can be produced by the stroma of tumor cells [12]. Under physiological conditions in the human body, IL-7 is found in very limited amounts, while the stromal cells produce IL-7 in approximately constant amounts, unaffected by external stimuli [13]. An increase in circulating IL-7 levels has been observed in diseases associated with lymphopenia, such as HIV infections, idiopathic CD4^+^T lymphopenia, and autoimmune diseases [14]. IL-7 acts through the IL-7 receptor (IL-7R), which consists of two chains: Alfa (α) and Gamma (ϒ). The ϒ chain is found in all types of hematopoietic cells, while the α chain is mainly expressed in lymphocytes, enabling the development of T and B lymphocytes, respectively, naive and memory T cells [15]. IL-7 action is realized through two main signaling pathways: Jak-Stat and PI3K-Akt [6] (Fig. 1). Through these pathways, they have an impact on the development, survival, proliferation, differentiation, and maturity of immune cells such as T-lymphocytes, B-lymphocytes, and natural killer cells [16]. Some evidence suggests that IL-7 overexpression influences the development and progression of a variety of tumors. Furthermore, IL-7 messenger RNA (mRNA) has been found in many types of tumors, such as renal, colorectal, and central nervous system (CNS) [17]. IL-7 receptor (IL-7R) mRNA has been seen in a variety of tumor cells, including breast, colon, lung, renal, and CNS [18]. It is now known that IL-7 induces the proliferation of a variety of cancers, such as leukemia and lymphomas [17].

The increased aggressiveness and metastasis in carcinomas, including BC, is associated with epithelial-mesenchymal transition (EMT) as it allows cells to invade surrounding tissues and through the bloodstream to enable the establishment of metastasis. Shreds of evidence show that the PI3K/Akt signaling pathway is implicated in EMT [19, 20]. In addition, overexpression of Akt has been seen frequently in human cancers and is related to tumor metastasis [21]. The results of a previous study show that the IL-7δ5 variant supports BC cell proliferation as well as cell cycle progression through the activation of the PI3K/Akt pathway [17]. On the other hand, evidence has shown that the administration of IL-7/IL-7Rα-Fc inhibits tumor growth and prolongs survival in lung cancer by inducing afferent and efferent antitumor responses [22]. Furthermore, cytotoxic T lymphocytes reduce pulmonary metastatic sarcoma in mice when stimulated by IL-7 [23]. The possibility of different reactions of IL-7 in different tumor models was described in the previous study [13]. These contradictory findings indicate that more research is needed to determine whether IL-7 functions as a promoter or inhibitor of tumor growth, but they also highlight IL-7’s association with tumor development. However, there is a lack of information regarding the correlation between IL-7 serum levels and the histopathological characteristics of BC or the prognosis of the disease.

Thus, the aim of this study has been to observe if there is any elevation in the IL-7 serum level in the EIBC patients compared to the healthy control group. In addition, the correlation between the IL-7 serum level and tumor size, histological type, histological grade, lymphovascular invasion (LVI) and perineural invasion (PNI), metastatic lymph nodes, and molecular markers such as estrogen receptor (ER), progesterone receptor (PR), amplification of Her-2/neu, Ki-67 proliferation index, molecular surrogate subtypes, age, and menopausal status has been evaluated.

The data from this study will contribute to the discussion about the role of IL-7 in the development of BC, in particular, to evaluate the possibility of using IL-7 as a tumor biomarker and prognostic indicator in BC.

## Methods

This dual-center, cross-sectional, observational, and analytical study has been undertaken in the Clinic of Oncology, University Hospital Center Zagreb, Croatia, and the Department of Thoracic Surgery in the University Clinical Center of Kosovo in Prishtina, Kosovo, from June 2018 until December 2019. In order to determine the IL-7 serum level, blood samples (10 ml of venous blood) have been taken from 213 EIBC patients, of whom 100 from Kosovo University Hospital and 113 from Croatian University Hospital, prior to surgery and other oncological treatments such as chemotherapy, radiotherapy, endocrine therapy, or target therapy. On the other hand, the blood samples (10 ml of venous blood) were also taken also from the other 62 healthy participants, who composed the control group, of whom 32 were from Kosovo and the other 30 from Croatia. Blood samples categorized in tubes have been identifiable only by the numbers attributed to participants and have been stored frozen at temperatures of − 20 °C, in order to conduct the analysis later.

Patients have been selected as consecutive among women, aged 20–70 who are confirmed to have BC, by biopsy, operable, and without distant metastases. Patients with pathological conditions, such as acute and/or chronic inflammatory diseases, rheumatoid disease, and other malignancies occurring simultaneously; patients receiving immune-modulatory therapy; patients previously treated by surgery, chemotherapy, radiotherapy, target therapy, or endocrine therapy; and patients with dementia, or any other psychological disorders unable to willingly participate in the study, have been excluded. The control group has been composed of women aged 20–70, without breast tumors confirmed by ultrasound or mammography in the last 3 months, or any other confirmed malignancies, without any acute or chronic inflammatory disease, as well as those not receiving immune-modulatory therapy.

In this study, the IL-7 serum level has been measured using the “Sandwich” ELISA Immunoenzyme test, human IL-7 antibody, and Platinum ELISA using research tools from eBioscience Inc., located in San Diego, CA, USA. After the surgery (partial or modified radical mastectomy) with or without axillary lymph node dissection or sentinel lymph node biopsy (SLNB), the histopathological specimen examinations have been performed according to routine practice, and the histopathological and immunohistochemical features have been evaluated. Cancer staging has been conducted according to tumor node metastasis (TNM)-classification by the American Joint Committee on Cancer Classification (AJCC) [24]. The patients have been classified in accordance to their pathological characteristics, including tumor size, histological types, histological grade, LVI and PNI, metastatic lymph nodes, and molecular markers such as ER, PR, amplification of Her-2/neu, Ki-67 proliferation index, and molecular surrogate subtypes according to the St Gallen consensus criteria [25]. The data on age and menopausal status has been obtained from the patients as well.

IL-7 serum levels have been initially determined in all the study participants, including patients and the control group. Then, it was evaluated if there had been any difference in IL-7 serum levels between EIBC patients and the control group. After that, appropriate analyses have been done to see if there is any correlation between the IL-7 serum level of the patients and the histopathological characteristics of the tumor, as well as age and menopausal status. In addition, the potential difference in IL-7 serum levels between patients coming from Croatia and Kosovo has been evaluated.

After checking for normality of distribution, numerical variables have been presented as median and interquartile ranges. The differences in the distribution of the numerical variables have been analyzed with the Mann–Whitney *U* test and Kruskal–Wallis ANOVA test. Associations between numerical variables have been analyzed as Spearman rank correlation coefficients. Analyses have been performed with the statistical software SPSS-22.0. A *P*-value < 0.05 was considered statistically significant. The diagnostic accuracy and the optimal cut-point value for the IL-7 level between two groups have been obtained based on the value of the area under the ROC curve.

## Results

Characteristics of all subject variables considered in the two groups, Kosovo and Croatia, are shown in Table 1. The two groups of participants were balanced in terms of the clinical and pathological features of the tumors at the time of the study.

**Table 1:** Summary of all the variables considered in the study

Variable	Kosovo (*n* = 100)	Croatia (*n* = 113)	All (213)
Age			
< 40 years	12	11	23
≥ 40 years	88	102	190
Premenopausal	33	37	70
Postmenopausal	67	76	143
Histological type			
IDC (NST)	76	89	165
ILC	16	14	20
Other	18	10	28
Axillary involvement			
Yes	47	39	86
No	53	74	127
pN( +) status			
N0	53	75	128
N1	20	27	47
N2	17	9	26
N3	10	2	12
Stage			
I	19	58	77
II	54	43	97
III	29	10	39
Histological grade			
G1	3	10	13
G2	69	69	138
G3	28	34	62
Ki-67%			
< 20	41	49	90
> 20	59	64	123
ER status			
ER +	77	92	169
ER −	23	21	44
PR status			
PR +	20	45	65
PR −	80	68	148
Her2/new status			
Her 2( +)	24	16	40
Her 2( −)	76	97	173
LVI status			
Positive	52	20	72
Negative	48	93	141
Metastatic lymph nodes			
Positive	47	38	85
Negative	53	75	128
Molecular subtypes			
Lum. A	35	42	77
Lum. B/Her2 neg	32	43	75
Lum. B/Her2 poz	12	8	20
Her2 positive	11	8	19
Triple negative	10	12	22
IL-7/median (25–75%)	66.1 (31.5–183.3)	54.7 (18.0–134.3)	

We examined the contribution of IL-7 to early invasive breast cancer. To this end, we analyzed serum levels of IL-7. The IL-7 serum level in EIBC patients was significantly higher than I control cases (*P* < 0.001) (Table 2).

**Table 2:** The distribution of the IL-7 serum level of the breast cancer patients and the controls

Variable	Breast cancer patients (*N* = 213)	Control cases (*N* = 62)	Mann–Whitney *U* test adjusted *z* = 9.23;
IL-7 serum level median (25–75%)	61.7 (24.3–152.6)	4.6 (2.7–12)	*P* < 0.001

**Fig. 2:**
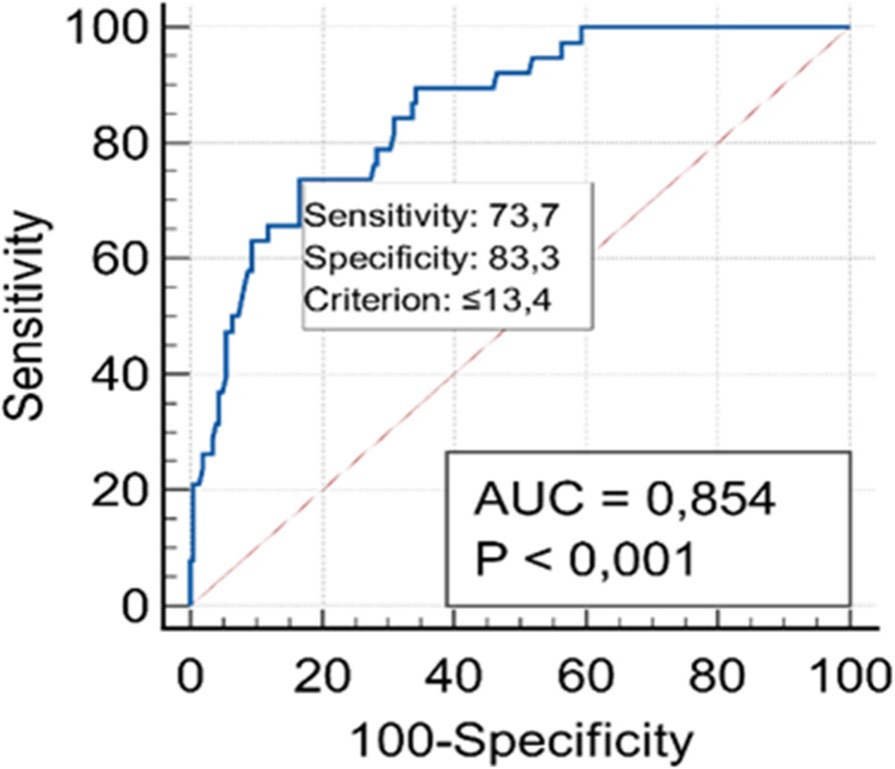
The standard ROC curve analysis of all patients and the control group. IL-7 level 14 has a sensitivity of 73.7% and a specificity of 83.3% for carcinoma, with an AUC of 0.854 (95% CI 0.803–0.896) at a cut-off value of 13.4 pg/ml

**Fig. 3:**
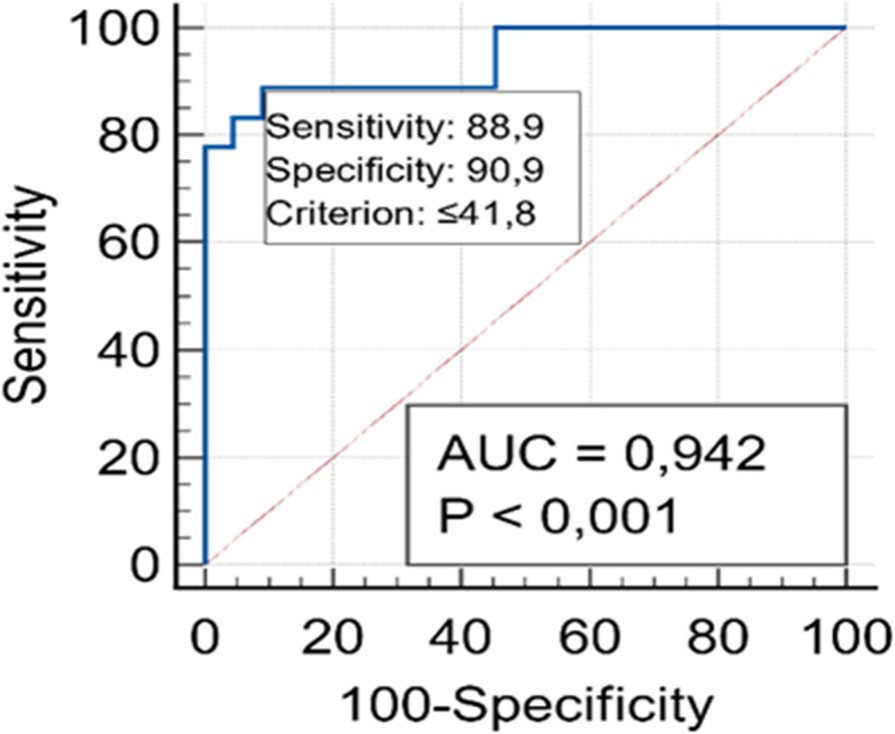
The ROC curve analysis of patients with high IL-7 values (≥ 99 pg/ml). IL-7 level 99 showed 88.9% sensitivity and 90.9% specificity for carcinoma. AUC was 0.942 (95% CI 0.819–0.991) at a cut-off value of 41.8 pg/ml

The diagnostic accuracy of a biomarker is a crucial factor. Accordingly, ROC curve analysis has been applied to analyze the diagnostic accuracy of measurements and optimal cut-point values for IL-7 level between verified EIBC and control groups (Figs. 2 and 3).

Regarding the EIBC patients, there have been no correlations between IL-7 serum level and tumor size, age, and Ki-67 (%) (Spearman’s *R* = 0.5, *P* = 0.4; Spearman’s *R* = 0.5, *P* = 0.6; and Spearman’s *R* = 0.3, *P* = 0.7, respectively) (Table 3).

**Table 3:** Correlation of the tumor size, age, and involved lymph node numbers with IL-7 serum levels

	Spearman’s coefficient	*P* value
Tumor size (mm)/IL-7	0.057	*P* = 0.4
Age/IL-7	0.024	*P* = 0.6
Ki-67/IL-7	0.028	*P* = 0.7

Patients with invasive lobular carcinoma (ILC) seem to have a lower IL-7 serum level as compared to other histological subtypes, and the difference is significant (*P* = 0.043). There have been no significant differences in IL-7 serum levels between different stages of the disease, molecular surrogate subtypes, and histological grades. Immunohistochemical expression or absence of molecular markers, such as PR, ER, amplification of Her-2/neu receptor, and proliferation index as measured by Ki-67, as well as LVI and PNI, have not been correlated with IL-7 serum level (Table 4).

**Table 4:** The distribution of the IL-7 serum level according to the tumor characteristics

Grouping variables	Categories of grouping variables (*n*)	IL-/7 Median (25–75%)	*P* value
Histological subtype	IDC (NST) (165)	65.25 (29.4–166,0)	Kruskal–Wallis ANOVA test value *H* (2.0) = 6.30; *P* = 0.043
	ILC (20)	18.5 (6.9–150,3)	
	Other (28)	74.6 (23.4–148.0)	
Axillar involvement	Yes (86)	70.9 (30.8–166.5)	Mann–Whitney adj z = 0.94; *P* = 0.348
	No (127)	58.8 (22.9–147.0)	
T-stage	T1 (103)	59.0 (22.8–144.1)	Kruskal–Wallis ANOVA test value *H* = 0.3; *P* = 0.86
	T2 (101)	64.0 (25.1–166.7)	
	T3 (9)	57.9 (26.9–182.0)	
Stage	I (77)	52.1 (19.2–134.3)	Kruskal–Wallis ANOVA test value *H* = 1.72; *P* = 0.42
	II (97)	63.6 (26–166.4)	
	III (39)	79.9 (23.9–169.3)	
Grade	I (13)	42.4 (11.6–174.1)	Kruskal–Wallis ANOVA test value *H* (2.213) = 1.14; *P* = 0.57
	II (138)	62.4 (29.1–166.1)	
	III (62)	62.4 (16.8–129.2)	
ER	Positive (169)	59.0 (23.9–159.3)	Mann–Whitney adj *z* = 0.53; *P* = 0.593
	Negative (44)	66.1 (20.0–168.2)	
PR	Positive (65)	40.5 (11.7–110.9)	Mann–Whitney adj *z* = 0.96; *P* = 0.334
	Negative (148)	66.7 (29.9–168.1)	
Her-2	Positive (40)	66.8 (26.6–140.0)	Mann–Whitney adj *z* = 1.42; *P* = 0.672
	Negative (172)	61.1 (23.4–166.0)	
Molecular surrogate subtypes	LumA (77)	71.1 (34.1–183.9)	Kruskal–Wallis ANOVA test value *H* (5.47) 4.0; *P* = 0.247
	LumB/HER2 neg (75)	51.1 (18.9–117.3)	
	LumB/HER2 pos (20)	52.6 (23.9–90.0)	
	Her-2 overexpression (19)	96.6 (43.9–225.3)	
	TN (22)	57.2 (17.9–151.0)	
LVI	Positive (85)	62.6 (34.7–166.3)	Mann–Whitney adj *z* = 0.7; *P* = 0.484
	Negative (128)	62.4 (23.1–156.0)	
PNI	Positive (24)	69.0 (37.9–166.8)	Mann–Whitney adj *z* = 0.87; *P* = 0.385
	Negative (80)	54.5 (22.9–158.1)	

There is no differentiation in the IL-7 serum level between premenopausal and postmenopausal patients, nor between patients coming from Croatia and Kosovo (Table 5).

**Table 5:** Distribution of the IL-7 serum level in patients from Kosovo and Croatia based on menopausal status

	*N*	IL-7/median (25–75%)	*P* value
Country	Kosovo (100)	66.1 (31.5–183.3)	Mann–Whitney adj *z* = 1.42; *P* = 0.156
	Croatia (113)	54.7 (18.0–134.3)	
Postmenopausal	Yes (144)	67.0 (25.3–166.4)	Mann–Whitney adj *z* = 0.56; *P* = 0.575
	No (69)	52.1 (20.3–137.3)	

## Discussion

A significantly higher IL-7 serum level in EIBC patients as compared to healthy control cases (*P* < 0.001) has been noticed. On the other hand, no link has been found between IL-7 serum levels and tumor size, lymph node metastasis, or tumor progression from well to poorly differentiated. The higher IL-7 serum level in BC patients can be explained by the production of different cytokines, including IL-7, by cancer cells [24], but the discontinuing increase of IL-7 serum levels during tumor growth and disease progression is controversial. Another reason for the increase of IL-7 serum levels at the start of tumor growth can be the counteraction of the immune system to the tumor, producing various cytokines, among them IL-7. However, as the tumor progresses in growth, metastasis in lymph nodes, or passes from well to poorly differentiated, the immune parameters fail and are replaced by other inflammatory cytokines, which support the tumor’s growth and progression [26]. It is known that the immune system has a key role in a mechanism of preventing the occurrence of cancer, but the immunity fails to control the tumor growth and progression due to strong defense mechanisms developed by the tumor [27–30]. In the tumor microenvironment, immune cells that are tolerant toward tumors, such as exhausted cytotoxic T lymphocytes, macrophages, and T helper cell type 2, can be found, as well as more myeloid-derived suppression cells (MDCS) and T regulatory cells (Treg), responsible for inhibition of effector immune responses [31, 32]. Cancer patients frequently have low T cell counts and, as a result, an immunosuppressive state [33]. Because IL-7 is responsible for the development, growth, and maturation of T lymphocytes [6], low level of T lymphocytes in patients with malignant diseases may be one reason for the increase in circulating IL-7 levels. The increase of the IL-7 serum level in the BC patients in this study does not correspond with the Iranian study, in which no elevation of the IL-7 serum level in the BC patients has been noticed. Moreover, their results show a higher level of IL-7 in the control group [12]. Furthermore, in the Iranian study, a higher level of IL-7 was seen in the serum of well-differentiated BC patients compared to patients with poor differentiation, while in our study there was no difference in the IL-7 serum level between patients with poor and well-differentiated tumors. The previous study has shown that the impact of the IL-7δ5 variant through the activation of Akt has a critical role in cell proliferation, apoptosis, angiogenesis, and metastasis [17], but in our study, there is no correlation between IL-7 serum level and lymph node metastasis, LVI, or PNI, and neither with tumor size has been noticed. Interestingly, a lower value of IL-7 has been observed in the serum of patients with ILC compared to patients with invasive ductal carcinoma (IDC) and other histological subtypes, and the difference has been significant (*P* = 0.043). The differences are already evident both histologically and clinically between the two main subtypes of BC, ILC, and IDC, which account for about 95% of all subtypes. Recent studies have also described marked differences in genomic levels between these two subtypes, while genetic level studies are still insufficient [34–36]. Given these differences, it can be probably assumed that the immune system’s response may be different in these two subtypes, specifically the production of different cytokines, in this case IL-7. Determining the risk factors that are associated with disease progression and metastasis is of great importance in treatment planning as well as in follow-up after treatment. Different surrogate subtypes of BC have different courses of the disease. The study done by Anwar et al. [37] found that patients with luminal A-like had better disease-free and overall survival as well as bone metastasis, while patients with non-luminal triple negative BC have a tendency to spread in the lungs and generally have a poor prognosis. But, in our study, there was no difference in IL-7 serum level between several molecular surrogate subtypes and in overexpression or not of molecular markers such as ER, PR, and Her-2/neu. No differentiation has been found in IL-7 serum levels between patients regarding age, as well as between patients coming from Croatia and Kosovo.

Menopause is not a factor that causes BC, but there are several risk factors associated with it. The longer exposure to estrogen lasts, the greater the chance of developing BC. In a previous study [38], there were several biological and clinical-pathological differences between premenopausal and postmenopausal BC patients. There was more pronounced heterogeneity and aggressiveness of the disease in the premenopausal group, with a higher rate of lymph node involvement and, as a result, a higher stage of the disease [38]. Molecular markers such as ER, PR, and Her2/neu, which also serve as prognostic factors, are distributed differently in premenopausal and postmenopausal BC patients, with a tendency for overexpression of the triple-negative cases in premenopausal ones [39]. Also, the rate of the cell’s poor differentiation is higher in premenopausal patients. Based on these data, BC tends to be more aggressive and with a worse prognosis in premenopausal women. However, no difference has been observed in the IL-7 serum level between premenopausal and postmenopausal patients.

Tumor markers have an important and ever-increasing role in all aspects of malignant disease care. The detection of a tumor marker can be done in tissue or in various body fluids, including serum, pleural fluid, or ascites. Clinical use may be done for screening or early detection, completion of diagnosis, determination of prognosis, monitoring of the effectiveness of therapy, and follow-up of disease and recurrence. As tumor markers, there can be different substances: surface antigens, different cytoplasmic proteins, hormones, enzymes, different oncogenes, and their products. Nowadays, when personalized treatment is becoming more and more important, the definition of genetic biomarkers has a special importance in the treatment of BC. The TNM staging system has flaws, so in the 8th edition of the AJCC, ER, and PR expression, as well as Her2/neu, as biological markers, have been included to determine clinical prognostic staging more accurately. In addition, the use of genetic biomarkers is recommended when it is possible. Three of them (*ESR1*, *PGR*, and *KIF2C*) have already been included in the AJCC recommendations [40]. The known biological functions and pathways that are encoded by certain biological markers enable their use to predict clinical outcomes as well as the evaluation of therapeutic possibilities. In another study, Zhou et al. [41] examined gene expression data from the TCGA database of BC patients. Analyses identified several important differentially expressed genes, of which *ABC*_*3*_, *CCL*_*22*_, *FOXJ*_*1*_, *IL*_*1*_*RN*, and *MAP2K6* were factors associated with good prognosis, while *KCNIP*_*3*_ and *MRPL*_*13*_ were associated with poor prognosis of the BC patients.

This study has some limitations. First, although the criteria for inclusion of the participants in this study were clear and meticulously respected, it is still impossible to completely avoid that all participants in the study did not have any additional malignant or unconfirmed inflammatory disease. Second, except for age and menopausal status, other clinical data on patients is lacking. The lack of data on parity, body mass index, hormone replacement therapy, alcohol and tobacco use, diet, and physical activity limits our ability to have a clearer picture of the risk factors that affect the occurrence of BC, and at the same time, it limits our ability to assess the eventual correlation between IL-7 and these risk factors.

## Conclusion

Observing the significant increase in the IL-7 serum level in the EIBC patients as compared to the healthy control group, it can be assumed that IL-7 can be used as a potential indicator for the diagnosis of BC. The lack of correlation with tumor size, lymph node metastasis, and all other histopathological characteristics of the tumor, maybe due to the production of IL-7 by immune system cells and not by tumor cells, questions its use as a prognostic indicator. The re-evaluation of the IL-7 serum levels of the patients participating in this study in the near future, as well as during follow-up, would enable assessing the correlation between IL-7 serum level and potential disease recurrence and, at the same time, evaluate the possibility of using it as an independent prognostic factor. With a reconfirmation of the correlation between IL-7 and BC through a study in a larger number of patients as well as the eventual correlation with the recurrence of the disease, the investigation of IL-7 as a proinflammatory cytokine could be tested as a poor prognostic factor and could help us to stratify the risk of recurrence. On the other hand, it could be used to follow up on the patients after oncological treatment. However, further studies with an even larger number of participants should be performed in order to determine the origin of IL-7, whether it is from BC cells or from the immune system cells, as well as to see the correlation between IL-7 serum levels and other interleukins.

## Data Availability

The datasets generated and/or analyzed during the current study are available in the [Harvard Dataverse] repository, “The Role of Serum Interleukin-7 level as Biological Marker in Breast Cancer” (https://doi.org/10.7910/DVN/YG3OLR), Harvard Dataverse, DRAFT VERSION, UNF:6:3:dNeDrCcWjX4fC5DDUlluA.

## Abbreviations

EIBC: Early invasive breast cancer
BC: Breast cancer
IL-7: Interleukin-7
IL-7R: Interleukin-7 receptor
IL1RN: Interleukin-1 receptor antagonist
PI3K: Phosphoinositide 3 kinase
JAK-1: Janus kinases-1
JAK-3: Janus kinases-3
STAT-5: Signal transducers and activators of transcription-5
CNS: Central nervous system
CCL22: C–C motif chemokine ligand 22
EMT: Epithelial mesenchymal transition
ESR1: Estrogen receptor 1
PI3K/Akt: Phosphoinositide 3-kinaze/protein kinase B
Akt: Protein kinase B (serine/threonine-specific protein kinase)
ABC_3_: ATP-binding cassette protein 3
FOXO1: Forkhead box protein O1
FOXJ1: Forkhead box J1
PR: Progesterone receptor
PGR: Progesterone receptor (GeneCards)
KIF2C: Kinesin family member 2C
KCNIP3: Potassium voltage-gated channel interacting protein 3
ER: Estrogen receptor
MDCS: Myeloid-derived suppression cells
MAP2K6: Mitogen-activated protein kinase kinase 6
MRPL13: Mitochondrial ribosomal protein L13
Treg: T regulatory cells
AJCC: American Joint Committee on Cancer System
TNM: Tumor node metastasis
NST: No special type
IDC: Invasive ductal carcinoma
ILC: Invasive lobular carcinoma
LVI: Lympho-vascular invasion
PNI: Perineural invasion
ASCO: American Society of Clinical Oncology
WHO: World Health Organization
IARC: International Research Agency
SEER: Surveillance, Epidemiology, and End Results
NCI: National Cancer Institute
ENCR: European Network of Cancer Registries
SLNB: Sentinel lymph node biopsy
UHCZ: University Hospital Center Zagreb
FISH: Fluorescent in situ hybridization
CISH: Chromogenic in situ hybridization

## References

[1] Sung H, Ferlay J, Siegel RL, Laversanne M, Soerjomataram I, Jemal A (2021). Global cancer statistics 2020: GLOBOCAN estimates of incidence and mortality worldwide for 36 cancers in 185 countries. CA Cancer J Clin.

[2] Gonzalez H, Hagerling C, Werb Z (2018). Roles of the immune system in cancer: from tumor initiation to metastatic progression. Genes Dev.

[3] Kallio R, Surcel HM, Bloigu A, Syrjälä H (2001). Balance between interleukin-10 and interleukin-12 in adult cancer patients with or without infections. Eur J Cancer.

[4] Yeung YT, McDonald KL, Grewal T, Munoz L (2013). Interleukins in glioblastoma pathophysiology: implications for therapy. Br J Pharmacol.

[5] Surh CD, Sprent J (2008). Homeostasis of naive and memory T cells. Immunity.

[6] Kittipatarin C, Khaled AR (2007). Interlinking interleukin-7. Cytokine.

[7] Mazzucchelli RI, Warming S, Lawrence SM, Ishii M, Abshari M, Washington AV (2009). Visualization and identification of IL-7 producing cells in reporter mice. PLoS ONE.

[8] Link A, Vogt TK, Favre S, Britschgi MR, Acha-Orbea H, Hinz B (2007). Fibroblastic reticular cells in lymph nodes regulate the homeostasis of naive T cells. Nat Immunol.

[9] Onder L, Narang P, Scandella E, Chai Q, Iolyeva M, Hoorweg K (2012). IL-7-producing stromal cells are critical for lymph node remodeling. Blood.

[10] Di Carlo E, D’Antuono T, Pompa P, Giuliani R, Rosini S, Stuppia L, et al. The lack of epithelial interleukin-7 and BAFF/BLyS gene expression in prostate cancer as a possible mechanism of tumor escape from immunosurveillance. Clin Cancer Res. 2009;15:2979–87. 10.1158/1078-0432.CCR-08-1951 19366834

[11] Guimond M, Veenstra RG, Grindler DJ, Zhang H, Cui Y, Murphy RD (2009). Interleukin 7 signaling in dendritic cells regulates the homeostatic proliferation and niche size of CD4+ T cells. Nat Immunol.

[12] Bordbar E, Malekzadeh M, Ardekani MT, Doroudchi M, Ghaderi A (2012). Serum levels of G-CSF and IL-7 in Iranian breast cancer patients. Asian Pac J Cancer Prev.

[13] Gao J, Zhao L, Wan YY, Zhu B (2015). Mechanism of action of IL-7 and its potential applications and limitations in cancer immunotherapy. Int J Mol Sci.

[14] Bolotin E, Annett G, Parkman R, Weinberg K (1999). Serum levels of IL-7 in bone marrow transplant recipients: relationship to clinical characteristics and lymphocyte count. Bone Marrow Transplant.

[15] Jiang Q, Li WQ, Aiello FB, Mazzucchelli R, Asefa B, Khaled AR (2005). Cell biology of IL-7, a key lymphotrophin. Cytokine Growth Factor Rev.

[16] Zarogoulidis P, Lampaki S, Yarmus L, Kioumis I, Pitsiou G, Katsikogiannis N (2014). Interleukin-7 and interleukin-15 for cancer. J Cancer.

[17] Pan D, Liu B, Jin X, Zhu J (2012). IL-7 splicing variant IL-7δ5 induces human breast cancer cell proliferation via activation of PI3K/Akt pathway. Biochem Biophys Res Commun.

[18] Cosenza L, Gorgun G, Urbano A, Foss F (2002). Interleukin-7 receptor expression and activation in nonhaematopoietic neoplastic cell lines. Cell Signal.

[19] Larue L, Bellacosa A. Epithelial-mesenchymal transition in development and cancer: role of phosphatidylinositol 3’ kinase/AKT pathways. Oncogene. 2005;24:7443–54. 10.1038/sj.onc.1209091 16288291

[20] Dupard-Julien CL, Kandlakunta B, Uppu RM (2007). Determination of epoxides by high-performance liquid chromatography following derivatization with N N-diethyldithiocarbamate. Anal Bioanal Chem.

[21] Yang J, Zeng Z, Peng Y, Chen J, Pan L, Pan D (2014). IL-7 splicing variant IL-7δ5 induces EMT and metastasis of human breast cancer cell lines MCF-7 and BT-20 through activation of PI3K/Akt pathway. Histochem Cell Biol.

[22] Andersson A, Srivastava MK, Harris-White M, Huang M, Zhu L, Elashoff D (2011). Role of CXCR3 ligands in IL-7/IL-7R alpha-Fc-mediated antitumor activity in lung cancer. Clin Cancer Res.

[23] Jicha DL, Mulé JJ, Rosenberg SA (1991). Interleukin 7 generates antitumor cytotoxic T lymphocytes against murine sarcomas with efficacy in cellular adoptive immunotherapy. J Exp Med.

[24] Giuliano AE, Connolly JL, Edge SB, Mittendorf EA, Rugo HS, Solin LJ (2017). Breast cancer-major changes in the American Joint Committee on Cancer eighth edition cancer staging manual. CA Cancer J Clin.

[25] Goldhirsch A, Winer EP, Coates AS, Gelber RD, Piccart-Gebhart M, Thürlimann B (2013). Personalizing the treatment of women with early breast cancer: highlights of the St Gallen international expert consensus on the primary therapy of early breast cancer 2013. Ann Oncol.

[26] Ravishankaran P, Karunanithi R (2011). Clinical significance of preoperative serum interleukin-6 and C-reactive protein level in breast cancer patients. World J Surg Oncol.

[27] Kim R, Emi M, Tanabe K (2007). Cancer immunoediting from immune surveillance to immune escape. Immunology.

[28] Matsushita H, Vesely MD, Koboldt DC, Rickert CG, Uppaluri R, Magrini VJ (2012). Cancer exome analysis reveals a T-cell-dependent mechanism of cancer immunoediting. Nature.

[29] Gross E, Sunwoo JB, Bui JD (2013). Cancer immunosurveillance and immunoediting by natural killer cells. Cancer J.

[30] Oleinika K, Nibbs RJ, Graham GJ, Fraser AR (2013). Suppression, subversion and escape: the role of regulatory T cells in cancer progression. Clin Exp Immunol.

[31] Hurwitz AA, Watkins SK (2012). Immune suppression in the tumor microenvironment: a role for dendritic cell-mediated tolerization of T cells. Cancer Immunol Immunother.

[32] Stewart TJ, Smyth MJ (2011). Improving cancer immunotherapy by targeting tumor-induced immune suppression. Cancer Metastasis Rev.

[33] ElKassar N, Gress RE (2010). An overview of IL-7 biology and its use in immunotherapy. J Immunotoxicol.

[34] Du T, Zhu L, Levine KM, Tasdemir N, Lee AV, Vignali DAA (2018). Invasive lobular and ductal breast carcinoma differ in immune response, protein translation efficiency and metabolism. Sci Rep.

[35] McCart Reed AE, Kutasovic JR, Lakhani SR, Simpson PT. Invasive lobular carcinoma of the breast: morphology, biomarkers and ’omics. Breast Cancer Res. 2015;17:12. 10.1186/s13058-015-0519-x PMC4310190 25849106

[36] Desmedt C, Zoppoli G, Gundem G, Pruneri G, Larsimont D, Fornili M (2016). Genomic characterization of primary invasive lobular breast cancer. J Clin Oncol.

[37] Anwar SL, Avanti WS, Nugroho AC (2020). Risk factors of distant metastasis after surgery among different breast cancer subtypes: a hospital-based study in Indonesia. World J Surg Onc.

[38] Nichols HB, Schoemaker MJ, Wright LB, McGowan C, Brook MN, McClain KM (2017). The premenopausal breast cancer collaboration: a pooling project of studies participating in the National Cancer Institute Cohort Consortium. Cancer Epidemiol Biomarkers Prev.

[39] Abubakar M, Figueroa J, Ali HR, Blows F, Lissowska J, Caldas C (2019). Combined quantitative measures of ER, PR, HER2, and KI67 provide more prognostic information than categorical combinations in luminal breast cancer. Mod Pathol.

[40] Kim J (2021). In silico analysis of differentially expressed genesets in metastatic breast cancer identifies potential prognostic biomarkers. World J Surg Onc.

[41] Zhou X, Xiao C, Han T (2020). Prognostic biomarkers related to breast cancer recurrence identified based on Logit model analysis. World J Surg Onc.

